# Effects of dust storm events on emergency admissions for cardiovascular and respiratory diseases in Sanandaj, Iran

**DOI:** 10.1186/s40201-014-0110-x

**Published:** 2014-08-06

**Authors:** Seyyed Jamal Aldin Ebrahimi, Leila Ebrahimzadeh, Akbar Eslami, Farzam Bidarpoor

**Affiliations:** Department of Environmental Health Engineering, Air Pollution Control, Kurdistan University of Medical Sciences, Sanandaj, Iran; Department of Environmental Health Engineering, Shahid Beheshti University of Medical Sciences, Tehran, Iran; Department of Environmental Health Engineering, Kurdistan University of Medical Sciences, Sanandaj, Iran

## Abstract

**Background:**

In recent years, increasing dust storms from western neighboring countries of Iran influenced western and central parts of the country. In this case, level of concentration of atmospheric particulate matter greater than 10 μm (PM_10_) remained higher for several days compared to the levels before the event. Accordingly, Suspended particulate matters of dust storms in western Iran have caused PM_10_ pollution in Sanandaj (capital of the Iranian province of Kurdistan) and other Iranian cities. The present study aimed to evaluate possible effects of dust storms on incidence of cardiovascular and respiratory diseases among residents of Sanandaj.

**Materials and methods:**

Dust storm events defined based on the Environmental Protection Administration and Meteorological Announcements, 21 March 2009 to 21 June 2010. Data related to the dust events associated with cardiovascular and respiratory diseases were obtained from the Center for Disaster and Emergency Medicine of Sanandaj, Iran. PM10 concentration and air quality data were obtained from air quality monitoring agency of Kurdistan Provincial Directorate of Environment Protection. Daily PM_10_ measurements were performed automatically according to β-ray absorption. Data were statistically analyzed using SPSS and Pearson's correlation coefficient. Also, linear regression model was used to investigate the relationship between variables.

**Results:**

The average PM_10_ levels during dust episodes (187 μg/m^3^) were significantly higher than the other days (48.7 μg/m^3^). In addition, correlation coefficient between PM_10_ level and number of cardiovascular and emergency service during dust events were equal to 0.48 (P <0.05) and 0.19 (P >0.05) respectively.

**Conclusion:**

Our findings showed significant increase in emergency admissions for cardiovascular and respiratory diseases during dust storms episode in Sanandaj. Although correlation between respiratory diseases and dust storm events were statistically insignificant (0.19), numbers of cardiovascular diseases were significantly correlated with dust storm events (0.48).

## Introduction

Arid or semi-arid environments, covering about 33% of the total world land area, are the major origin of dust events (World Meteorological Organization, 2013 #1). Dust storms occur when high winds at a threshold speed blow over low vegetation and soil areas that lack moisture content and are vulnerable to disturbance [1]. Increasing dust storms originating from western neighboring countries of Iran in recent years have influenced western and even central parts of Iran. Furthermore, PM_10_ (particulate matter greater than 10 μm) concentrations remained greater than before the episode for several days. These dust storms are associated with various environmental and socio-economic problems [2].

**Figure 1:**
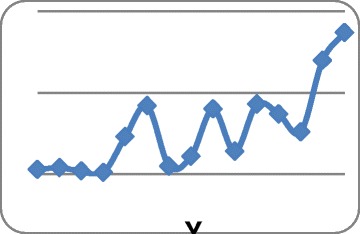
**Trend of dust storms in Sanandaj City over 1995–2009 period.**

**Figure 2:**
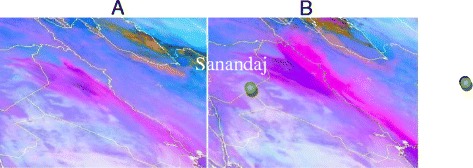
**Dust is observed in pink with a tone more intensive the higher the dust content in the atmospheric column.** **(A)** 16 June and 2008, **(B)** 17 June 2008, 10:30.

Suspended particulate matters of dust storms in the west of Iran cause PM_10_ pollution in Sanandaj and other cities (Figure 1) [3]. Amanollahi et al. showed that dust storms in this region were created by wind erosion in desert of Northern Saudi Arabia, Western Iraq, and Eastern Syria. Average values of PM_10_ in Sanandaj ranged 107 to 2,976 μg/m^3^ on 3 and 5 July 2009 were recorded as sever dust storm with maximum PM_10_ concentration of 5,616 μg/m^3^ at 2 a.m. on 5 July 2009 [4]. A significant high concentration of PM_10_ on 5 July, compared to 8 July 2008, 10:30 was shown in Figure 2a (left) and b (right) [5].

In 2010, air quality index of Sanandaj was interpreted as good (19%), moderate (57%), unhealthy for sensitive groups (15%), unhealthy (5%), very unhealthy (2%) and hazardous (2%) [6]. In some cases, measured concentrations of particles in the dust storms reached to more than 6,000 μg/m^3^ [7]. Although, World Health Organization defines 24-hour average concentration of PM_10_ for ambient air and its annual average as 50 and 20 μg/m^3^ respectively [8].

PM_10_ particles cause or aggravate a number of diseases and mortalities due to cardiovascular or respiratory conditions. People with cardiovascular or respiratory diseases such as congestive cardiovascular failure, coronary artery disease, asthma or chronic obstructive pulmonary disease and old people are more likely refer to emergency care centers, hospitalized or even die in some cases. Furthermore, cardiac irregularities and cardiovascular attacks were attributed to exposure to particles [9,10]. In a study conducted in China by Meng et al. reported increasingly frequent hospitalization for pneumonia during dust storms [11]. According to the WHO report, during the late 1990s, exposure to PM_10_ has caused the occurrence of 700 annual deaths due to acute respiratory infections in children under 4 years old in Europe [12]. With the increase of 100 μg/m^3^ in the 24 h average concentration of PM_10_, pneumonia and chronic obstructive pulmonary disease cases increased by 19% and 27%, respectively [13]. Ostro et al. (1999) found an association between PM_10_ and daily mortality in the Coachella Valley, a desert resort and retirement area east of Los angles (CA, USA) where coarse particles of geological origin typically comprise approximately 50–60% of PM_10_ and can exceed 90% during wind events [14]. The present study is aimed to evaluate possible effects of dust storms on the incidence of cardiovascular and respiratory diseases in Sanandaj during spring 2010.

## Methods

### Study region

**Figure 3:**
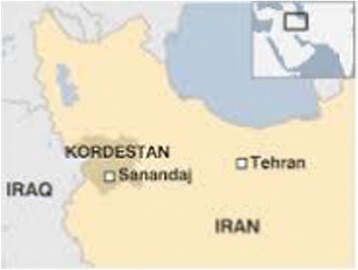
**Location of Sanandaj and Kurdistan province in Iran.**

Sanandaj, is located in western Iran (Figure 3). To assess the impacts of dust storms, we defined dust events based on Environmental Protection Administration and the Meteorological announcements from 21 March 2009 to 21 June 2010.

### Data on the incidence of diseases associated with dust events

Patients with cardiovascular and respiratory diseases who received medical services from the CDMME in Sanandaj during dust event days were considered for the study. According to the international classification of diseases, following codes are available for the two diseases: cardiovascular diseases with international code of 459–390, and respiratory diseases with the international code of 519–490. Therefore, the diseases which were in the above range (according to the international classification of diseases) were considered as cardiovascular and respiratory diseases.

### Data on PM_10_ concentration and air quality in Sanandaj

Sanandaj City possesses two pollution measurement stations (ECOTECH Company, Australia). These stations are capable of measuring air pollutants including carbon monoxide (CO), sulfur anhydride (SO_2_), particulate matter with 10 microns in diameter (PM_10_), nitrogen oxides (NO_X_) and ozone (O_3_). The first station is located in the State Environmental Protection Administration and the second in the campus of the health department of Kurdistan University of Medical Sciences. The monitoring stations were fully automated and provided daily readings of PM_10_ levels (by β-ray absorption). Measurement data were frequently calibrated by a private company.

### Statistical analysis

The diseases associated with dust storms and various concentrations of PM_10_ were analyzed using Microsoft Excel and linear regression. Equation of the regression line was used to indicate correlation between these two variables.

## Results

**Figure 4:**
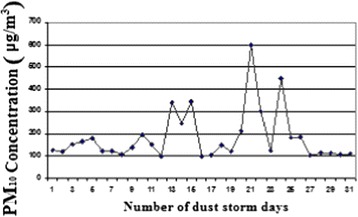
**The concentration of PM**_**10**_ **in the days of dust events in the spring.**

The average PM_10_ level during dust event days (187 μg/m^3^) was significantly higher than the comparison days (48.7 μg/m^3^) (Figure 4).

Table 1 shows air quality index for PM_10_ pollutant in spring. During 31 days of the season, Sanandaj city faced dust problem, so that air quality index was interpreted as follows: unhealthy within 16 days for sensitive groups, 8 days unhealthy, very unhealthy for 2 days and 5 days as unsafe.

**Table 1:** **Description of the air quality index for the days of spring for PM**_**10**_ **pollutant**

**Unsafe**	**Very unhealthy**	**Unhealthy**	**Unhealthy for sensitive groups**	**Healthy**	**Good**
5	2	8	16	43	19

Figure 4 shows number of days with high level of PM_10_ in spring. The number on pollutant days on March-April, April-May and May-June were 7, 8 and 16 days, respectively.

Minimum, maximum, and average concentrations of PM_10_ during dust event days in spring are presented in Table 2. Average concentration of this pollutant in stormy days is 187 μg/m^3^. Minimum and maximum 24-hour concentrations of PM_10_ were equal to 100.4 and, 599.6 μg/m^3^, respectively during May-June, 2010.

**Table 2:** **The maximum, minimum and, average daily levels of PM**_**10**_ **on stormy days of spring**

**Season**	**Maximum g/m** ^**3**^ **μ**	**Minimum μg/m** ^**3**^	**Average μg/m** ^**3**^	**Standard deviationα ≈ 0.95**
Spring	6/599	4/100	187	2/115

**Figure 5:**
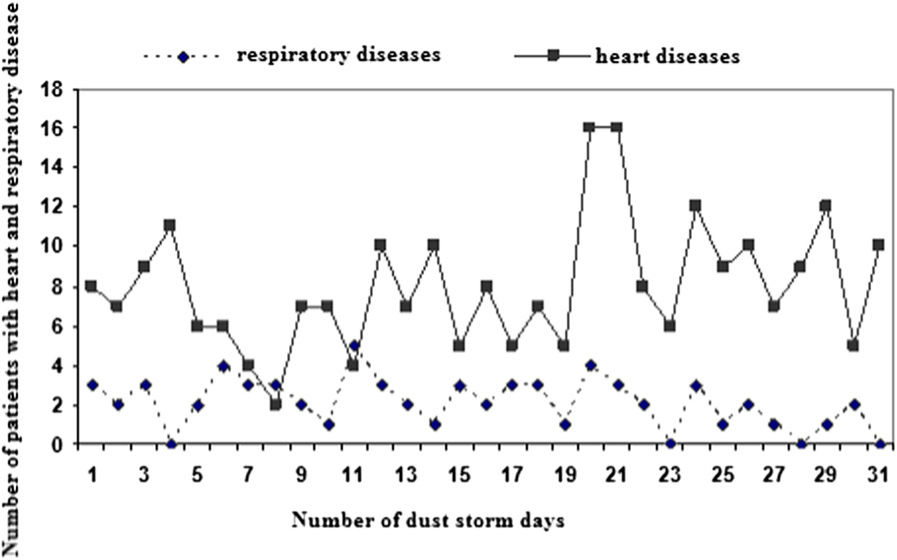
**The number of patients with cardiovascular and respiratory diseases received medical emergency services on dust events in spring.**

Figure 5 shows the number of patients with cardiovascular and respiratory problems received medical services from CDMME in Sanandaj during dust events days. Nuumber of cardiovascular diseases is more than the number of respiratory diseases. This is why cardiovascular diseases are the primary cause of mortality in Sanandaj and thereby a high number of cardiovascular patients compared to patients with respiratory diseases. Furthermore, comparison of Figures 3 and 4 reveals possible correlation between the numbers of patients with respiratory and cardiovascular diseases who received medical services from the CDMME with the increase in PM_10_ concentration. Linear trend of cardiovascular diseases is more consistent with the linear trend of PM_10_ concentration (Figure 4).

**Figure 6:**
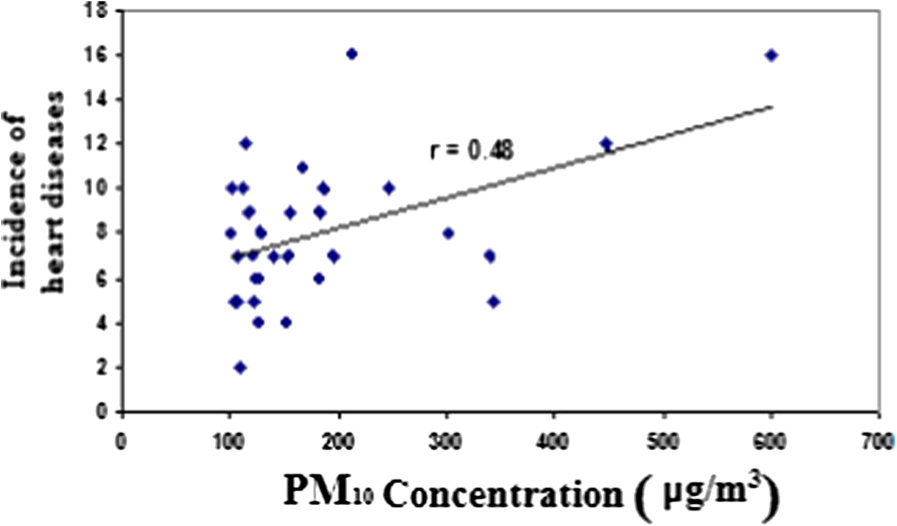
**The concentration of PM**_**10**_ **and the incidence of cardiovascular diseases on dust events.**

Linear regression model was used to investigate the relationship between variables. According to Figure 6 and data from the CDMME and statistical analysis, correlation coefficient between PM_10_ level and number of cardiovascular patients received emergency service on dust events were equal to 0.48 (P < 0.05) which indicates a significant positive correlation between these two variables.

**Figure 7:**
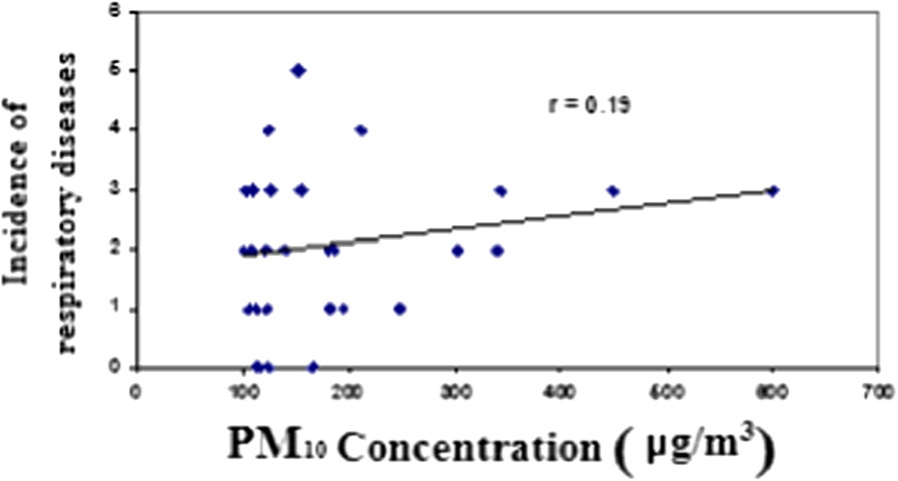
**The concentration of PM**_**10**_ **and the occurrence of respiratory diseases on dust events.**

The correlation coefficient between concentration of PM_10_ and number of respiratory patients receiving medical emergency services on dust events days was 0.19 (P > 0.05) (Figure 7). This indicates no significant linear correlation between these two variables.

Table 3 shows equations of the regression line and correlation coefficients between concentration of PM10 and the occurrence of respiratory and cardiovascular diseases. Our results showed 100 μg/m^3^ increase in PM_10_ concentration resulted in 1.351% and 0.021% increase in cardiovascular and respiratory diseases, respectively.

**Table 3:** **The correlation between the concentration of PM**_**10**_ **and the incidence of cardiovascular and respiratory diseases**

**Correlation coefficient**	**Regression line equation**	**Title**
0.48	y = 0.0135x + 5.5029	Cardiovascular diseases
0.19	y = 0.0021x + 1.7	Respiratory diseases

## Discussion

Average PM_10_ level during dust event days (187 μg/m^3^) was significantly higher than the comparison days (48.7 μg/m^3^). During storm event days other pollutants related to combustion (SO_2_, NO_2_, CO) were almost constant; therefore, any effects due to the occurrence of dust storms can be attributed to an increase in PM_10_ concentration.

Kwon et al. showed that during dust storms PM_10_ concentration from geological sources reached 50% to 60% and sometimes to 90% [15]. Several studies have shown that in dust storms, effect of fine particles is higher than coarse particles [16-18]. However, Dockery et al. found no difference between the impact of fine and coarse particles [19]. Castillejos et al. conducted a daily time-series analysis of mortality in relation to measurements of PM2.5, PM10, and PM10-2.5 in south western Mexico City in the years 1992–1995, they found that the effect of coarse particles was stronger for respiratory diseases than for total mortality, cardiovascular diseases, or other non injury causes of death [20].

Hefflin et al. investigated the effect of dust storms on the number of emergency referrals in one of the south-eastern states of Washington. During the dust storm events, 24 h PM10 concentrations in two consecutive days were more than 1000 μg/m^3^. Numbers of patients with bronchitis referring to the center were about 3.5% per each 100 μg/m^3^ increase in PM10 concentration [21]. According to the results of the present study, for each 100 μg/m^3^ increase in the PM10 concentration, 1.35% and 0.021% increase in the incidence of cardiovascular and respiratory diseases was observed respectively.

Pan et al. found a significant correlation between the dust storms and increased hospital admissions. The most common diseases during stormy days were pharynx, larynx, cornea, nasal and cervical inflammations [22].

Yang et al. investigated the effect of dust storms on the occurrence of cerebrovascular accident, 7 days before and 7 days after the dust storms events in Taiwan during 1996–2001. They found a significant difference between the storms events and the incidence of stroke 3 days after the events. However, no statistically significant difference was observed between the storm events and the incidence of cardiovascular attacks 3 days after the events [11].

Chiu et al. studied possible association between dust storms and hospital admissions of chronic obstructive pulmonary diseases 7 days before and after the dust storms events in Taiwan during 1996–2001. They found no significant difference between storm events and the incidence of chronic obstructive pulmonary diseases [23].

Chen et al. studied effect of dust storms on hospital admissions of cardiovascular diseases, 7 days before and 7 days after the dust storms events in Taiwan during 1996–2001. No significant differences were found between storms events and cardiovascular conditions [24].

## Conclusions

Occurrence of dust storms could increase the probability of cardiovascular and respiratory diseases in Sanandaj. Although the correlation between respiratory diseases and the dust storms was not statistically significant, a statistically significant correlation was observed between cardiovascular diseases and dust storms events.
